# Optimizing a Test Method to Evaluate Resistance of Pervious Concrete to Cycles of Freezing and Thawing in the Presence of Different Deicing Salts

**DOI:** 10.3390/ma9110878

**Published:** 2016-10-28

**Authors:** Chehong Tsang, Medhat H. Shehata, Abdurrahmaan Lotfy

**Affiliations:** 1 Department of Civil Engineering, Ryerson University, Toronto, ON M5B 2K3, Canada; chehong.tsang@ryerson.ca; 2 Lafarge Canada Inc., 6509 Airport Road, Mississauga, ON L4V 1S7, Canada; abdurahman.lotfy@lafargeholcim.com

**Keywords:** pervious concrete, salt scaling, freezing and thawing, test methods

## Abstract

The lack of a standard test method for evaluating the resistance of pervious concrete to cycles of freezing and thawing in the presence of deicing salts is the motive behind this study. Different sample size and geometry, cycle duration, and level of submersion in brine solutions were investigated to achieve an optimized test method. The optimized test method was able to produce different levels of damage when different types of deicing salts were used. The optimized duration of one cycle was found to be 24 h with twelve hours of freezing at −18 °C and twelve hours of thawing at +21 °C, with the bottom 10 mm of the sample submerged in the brine solution. Cylinder samples with a diameter of 100 mm and height of 150 mm were used and found to produce similar results to 150 mm-cubes. Based on the obtained results a mass loss of 3%–5% is proposed as a failure criterion of cylindrical samples. For the materials and within the cycles of freezing/thawing investigated here, the deicers that caused the most damage were NaCl, CaCl${}_{2}$ and urea, followed by MgCl${}_{2}$, potassium acetate, sodium acetate and calcium-magnesium acetate. More testing is needed to validate the effects of different deicers under long term exposures and different temperature ranges.

## 1. Introduction

Pervious concrete is a special type of concrete with an open structure that allows water to drain through. It is typically used in parking lots and other low volume pavements as a stormwater management tool by reducing runoff. Due to the porous nature of pervious concrete, its strength is generally lower than that of conventional concrete. The design of pervious concrete can be quite difficult as minor changes in the cement content, water-to-cement ratio, or aggregate gradation can have significant impact on strength and/or permeability.

Pervious concrete in cold weather presents additional challenges. Freezing and thawing cycles, in the presence of deicers, can cause severe damage to pervious concrete [1]. While damage due to freezing and thawing cycles in the presence of salt, or salt scaling, has been studied for many years in conventional concrete [2], there is a need for a standard test method to evaluate the resistance of pervious concrete to freezing and thawing cycles in the presence of deicing salts.

In conventional concrete, cycles of freezing and thawing in the presence of deicing salts results in the removal of chips from the surface of the concrete. As this phenomenon has been studied for many years, standard test methods, such as the ASTM C672, are used to evaluate the resistance of conventional concrete to this type of damage. In this test, a deicing solution is ponded on the top surface of a concrete slab. The slab then undergoes freezing and thawing cycles. The damage is determined visually and also by determining the mass of material removed from the surface.

While a number of mechanisms have been proposed over the years [3,4,5] suggesting that a physical mechanism is behind the failure due to freezing and thawing in the presence of salts in conventional concrete, it is important to consider the effects of long term exposure to deicing solutions. The effects of different types of decing salts on conventional concrete have been studied by a number of researchers. In these studies, a number of deicers are often compared against each other by exposing the concrete to freeze-thaw cycles, wetting/drying cycles, or submerging samples in a deicing solution at various temperatures.

The damaging effects of calcium chloride (CaCl_2_), magnesium chloride (MgCl_2_), and CMA has been reported in a number of earlier studies [6,7,8,9]. It was found that through wetting/drying cycles, CaCl_2_, MgCl_2_, and CMA can cause significant damage in terms of mass loss and a reduction in stiffness, particularly at higher concentrations [6]. In the same study, it was found that exposure to NaCl did not cause any significant negative effects on conventional concrete. The damaging effect of both CaCl_2_ and MgCl_2_ is often attributed to the formation of calcium oxychloride (3CaO·CaCl_2_·15H_2_O) [7,8]. While calcium oxychloride was reported to form quickest between 4 °C and 10 °C, damage still occurred through wetting/drying cycles at higher temperatures [6]. Long term exposure to CMA at high concentrations was found to cause severe deterioration of the cement matrix but can be at least partially mitigated through the use of slag as partial replacement of Portland cement [10]. The damage cause by CMA may be due to the formation of calcium acetate hydrate identified using X-ray diffraction analysis [11].

Acetates are often used as an alternative to conventional deicing salts as the chloride-based deicers can cause corrosive damage to reinforcing steel. The effects of potassium acetate on salt scaling had been studied by Wang et al. [12]. It was found that potassium acetate had caused only minor scaling at a 13.6% concentration. By contrast, NaCl (at 13.3%) and CaCl_2_ (at 9.5%) exhibited more severe scaling under the same testing conditions. However, the use of acetate based deicers can accelerate alkali silica reactions (ASR) if reactive aggregates are used [13]. Additionally, concretes made with aggregates with a high proportion of microfines suffered deterioration of the paste when exposed to potassium acetate [14].

In terms of resistance of pervious concrete to the damaging effects of freezing and thawing cycles in the presence of salts, a number of researchers have attempted to carry out tests using various lengths of freezing/thawing cycles, range of temperatures, different deicers, and different exposures [15,16,17,18]. While numerous mechanisms have been suggested for salt scaling in conventional concrete [3,4,19] as discussed above, there is no extensive published data for mechanisms of salt scaling in pervious concrete.

Mata [18] used the freeze-thaw cycle described in ASTM C672 with a freezing period of 16–18 h at −18 °C and a thawing period of 6–8 h at 23 °C. Since a deicing solution cannot be ponded on the top surface as described in ASTM C672, the bottom half of the discs were submerged in a 1% by mass CaCl_2_ solution. Both mixture types that did not contain any fine aggregate failed after eight cycles, even with high dosages of air entraining admixture. Damage was assessed through mass loss and change in dynamic modulus. The inclusion of fine aggregate and higher dosage of air entraining admixtures in the mix was found to enhance the durability.

Cutler et al. [15] tested the resistance to freezing and thawing in the presence of deicers of pervious concrete under both saturated and drained conditions. In the saturated conditions, samples were completely submerged in solution during testing. In the drained test method, samples would be submerged initially, but the solution would be drained prior to freezing. In both cases, the temperature varied between 10 °C and −18 °C. The change in temperature from the high end to the low end and vice versa took 2 h each, without holding the temperature at either the maximum or minimum, resulting in a total cycle length of only 4 h. Three different deicers were tested at a concentration of 9%, along with distilled water with no deicers. The deicers tested were calcium chloride (CaCl_2_), sodium chloride (NaCl), and calcium magnesium acetate (CMA). All samples used for this study were 100 mm cubes cured for 28 days in a humidity room prior to testing. The level of damage was assessed by measuring the mass loss and the compressive strength at regular intervals. The authors concluded that CaCl_2_ caused the most damage, followed by NaCl, and CMA. They had also found that the saturated test caused more damage than the drained test.

Anderson et al. [16] tested a longer cycle that ranged from 25 °C for 8 h in laboratory air to −20 °C for 16 h in a freezer . Samples were submerged in solution during the last hour of the thawing phase and drained prior to freezing. The authors claimed that this would be the case for a pervious concrete pavement under service conditions. NaCl was the only deicer that was studied at various concentrations (2%, 4%, 8%, and 12% by mass) as well as a set of control samples exposed to freezing and thawing in water without NaCl. All samples used were cylinders with a diameter of 100 mm by 200 mm height. The mass loss of each sample was measured at the end of every 10 cycles until the samples failed at 15% mass loss or when the sample reached 100 freeze-thaw cycles. The results indicated that a salt concentration of 4% and 8% caused the most salt scaling damage, with the damage resulting mainly from the scaling of the cement paste, with little damage to the aggregate. In conventional concrete, a “pessimum” concentration of 3% by mass, has been reported in literature [20]. It was also shown that the damage originated at the bottom of the sample where the level of saturation would be highest due to the draining of the solution. The solution would move towards the bottom of the sample as it drains and would likely freeze before leaving the sample completely. At lower and higher concentrations, 2% and 12% NaCl, very little damage was observed. It is likely that at high concentrations, the solution may take longer to freeze completely resulting in a reduced amount of time in a frozen state [16].

In this paper different test conditions were investigated to reach an optimized test that produces quantifiable and repeatable damage in pervious concrete samples due to freezing and thawing cycles in the presence of salts. The test involves exposing the pervious concrete samples to cycles of freezing and thawing in the presence of deicers. To optimize the test, different test parameters were tested for their effect on the rate and level of deterioration of the tested samples. The tested parameters included (1) sample shape and size; (2) conditions of the samples in terms of brine saturation; and (3) length of the freezing/thawing cycles. Finally, the optimized test was evaluated for its capacity to differentiate between different deicers.

## 2. Materials and Methods

### 2.1. Materials and Pervious Concrete Mix

**Figure 1:**
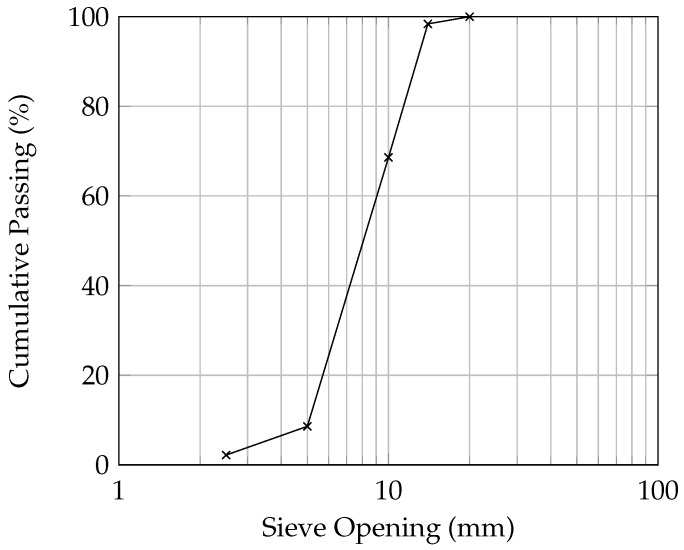
Grain size distribution curve of 14 mm limestone.

General Use (GU) Portland cement along with Granular Ground Blast Furnace Slag (GGBFS) was used in the production of pervious concrete samples for all experiments (Table 1). The mixture also contained natural limestone coarse aggregate without any additional fine aggregate. The gradation for the 14 mm limestone aggregate is provided in Figure 1. One mix design, with an average porosity of 25% was used with varying sample geometry for various tests. General mix design parameters are provided in Table 2. GGBFS was used in the mixture to reflect the industry practice in Ontario, Canada.

### 2.2. Sample Preparation

**Figure 2:**
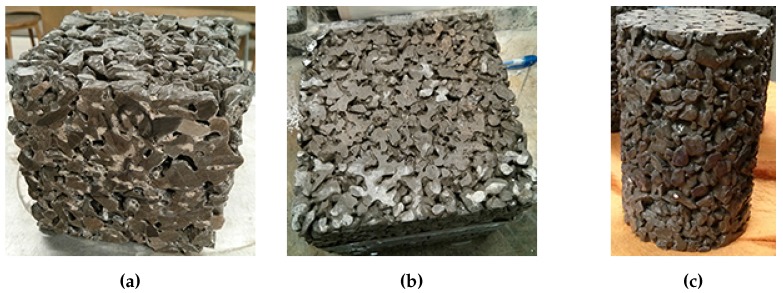
Various sample geometries used in the different salt scaling tests. (**a**) 100 mm cube; (**b**) 150 mm cube; (**c**) 100 mm diameter by 150 mm height cylinder.

Three different shapes of samples were prepared: (a) 100 × 100 × 100 mm cubes; (b) 150 × 150 × 150 mm cubes and; (c) 100 mm diameter × 150 mm height cylinders (Figure 2). The 100-mm cube samples were prepared by casting a 100 × 100 × 300 mm beam and saw cut into three cubes. The ends of the cubes were then ground flat using an end grinder. The 150-mm cubes were prepared in individual steel moulds. The cylinder samples were cast in 100 mm diameter × 200 mm height cylinder moulds. The ends were then saw cut and the ends ground flat to a final height of 150 mm. The ends of the samples were ground flat to enable compressive strength testing and to allow uniform depth of brine solution while samples were under the freezing/thawing testing. All samples were demoulded after 24 h and cured in a curing room at 100% relative humidity and room temperature for 28 days prior to testing.

### 2.3. Freezing/Thawing Cycles and Testing Conditions

The test for conventional concrete must be modified since a deicing solution cannot be ponded on the top surface of a pervious concrete slab. Three different freezing/thawing cycles were adopted in this program where the difference between them was mainly the duration of the freezing and thawing periods. The cycles are named in this paper according to their total duration; i.e., 12-h cycle, 16-h cycle and 24-h cycle. In addition, the depth of the brine solution varied from one test to another. The ponding of the brine solution would be representative of a pervious concrete pavement clogged with debris. The drained condition, in which samples were tested without ponding would be expected of a properly functioning pervious concrete pavement. The details of the testing conditions are described in the coming paragraphs. A summary is provided in Table 3 which lists the conditions of the cycles as well as the standard cycle used for conventional concrete, for the purpose of comparison.

#### 2.3.1. 12-h Cycle Under Drained Condition (Method 1)

**Figure 3:**
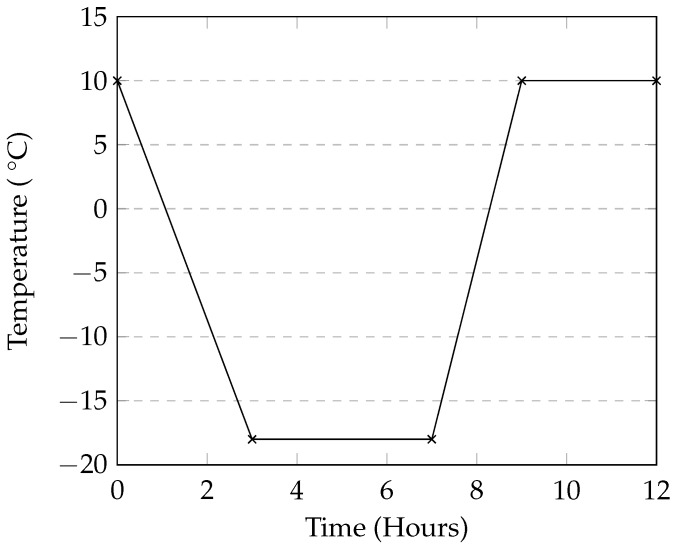
Temperature profile in the 12-h cycle.

For all tests, a calcium chloride solution at 4% concentration by mass was used. Eighteen 100 mm cubes and nine cylinders were used in this this test. Before the start of each cycle, samples were submerged in the CaCl_2_ solution for 30 min before being removed and transferred to a testing container. The samples were placed on plastic grid placed at the bottom of the container in order to minimize the area of contact between the container and the bottom of the samples. The grid also allowed the solution to drain out of the sample without pooling at the bottom. Each container was covered with plastic wrap in order to minimize evaporation. The containers were then covered and placed into an automatic environmental chamber where the temperature varied from 10 °C to −18 °C as shown in Figure 3. This temperature range was selected as a starting point based on the work by Cutler et al. [15].

After every 4 cycles, during the last two hours of the thawing period, the samples were removed from the environmental chamber and washed. Each sample is placed under a gentle stream of water to help remove any scaled off material trapped within the sample. Each sample was washed for 30 to 60 s depending on the level of damage or scaling. Following the wash, samples were placed on an absorbent cloth and left to dry for 30 min at room temperature. After drying for 30 min, the mass of the sample was determined and recorded. Compressive strength testing was carried out at the end of 16, 32, and 52 cycles on six cubes and three cylinders. The testing was concluded at the end of 52 cycles, close to the number of cycles used for evaluating salt scaling in conventional concrete according to ASTM C672.

#### 2.3.2. 12-h Cycle under Partially Saturated Condition (Method 2)

Nine cylinder samples were used for this test where the samples were submerged in 50 mm of the CaCl_2_ solution, while freezing and thawing. The cycles and washing procedure are the same as in Method 1. Three cylinders were removed for compressive strength testing at the end of 16, 32, and 52 cycles.

#### 2.3.3. 16-h Cycle under Partially Saturated Condition (Method 3)

**Figure 4:**
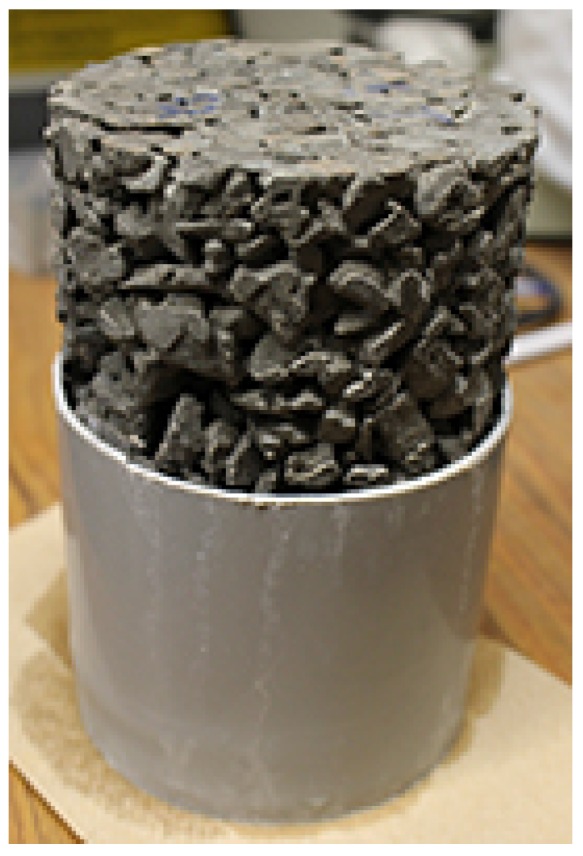
Pervious concrete cylinder partially saturated using a mould with a height of 75 mm.

**Figure 5:**
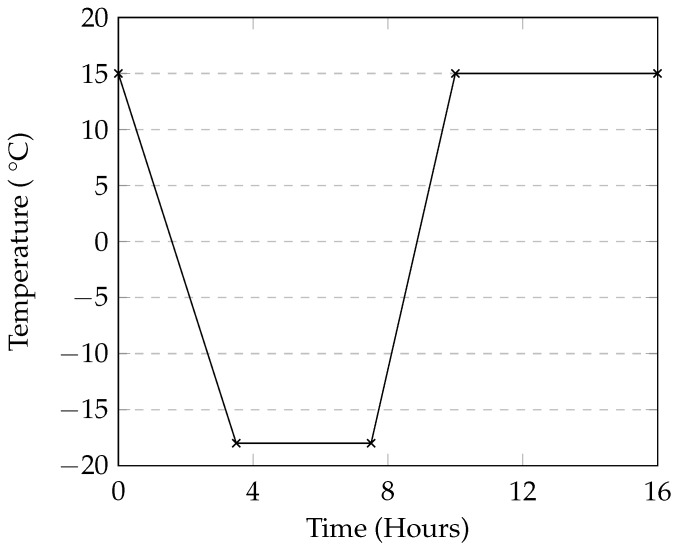
16-h temperature cycle.

Nine cylinder samples were each placed into individual plastic cylinder moulds with a diameter of 100 mm and a height of 75 mm (Figure 4). After the sample is placed in the mould, CaCl_2_ solution was poured in from the top of the sample until it overflowed out the top of the mould. Each mould was tapped gently to dislodge any remaining air bubbles and the solution was topped off once more and allowed to overflow. The use of the moulds minimized the amount of solution required to submerge the samples to ensure complete freezing and thawing of the solution. The samples were then placed in containers and the top of the container was covered with a plastic wrap prior to being placed in an environmental chamber. The cycle duration was 16 h with a temperature range varied from 15 °C to −18 °C with the cycle as shown in Figure 5. The increased thawing temperature was to ensure complete thawing of the solution; Results from the previous cycles suggested that the samples did not thaw completely. This will be presented later in this paper.

The samples were removed every 6 cycles for mass measurements using the same procedure, and to replace the solution. At the end 18, 36, and 54 cycles, three samples were tested for compressive strength. Testing was concluded at 54 cycles rather than 52 cycles due to samples being removed only once every 6 cycles.

#### 2.3.4. 24-h Cycle under Partially Saturated Condition (Methods 4, 5 & 6)

**Figure 6:**
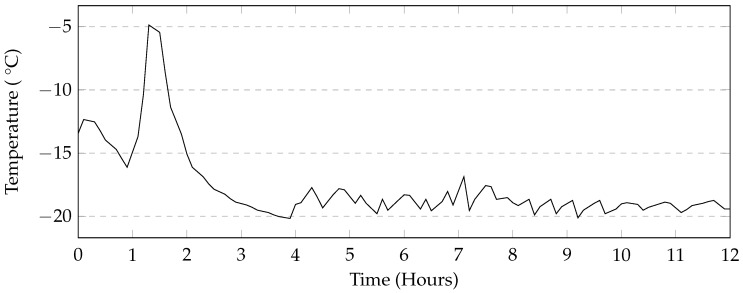
Typical air temperature profile of the freezer; initial peak caused by opening of the door; Second peak is due to the defrost system of the freezer.

**Figure 7:**
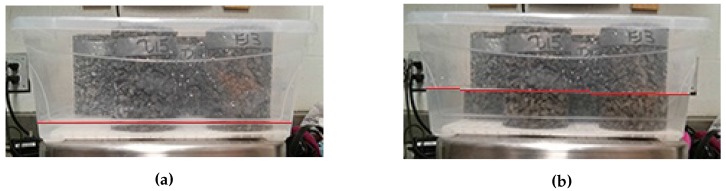
Containers filled with two levels of deicing solution. (**a**) 10 mm; (**b**) 50 mm.

Based on the limited amount of damage observed in the previous three cycles, a 24-h cycle was adopted (Method 4). The cycle consisted of 12 h of freezing at −18 °C, and 12 h thawing at 21 °C. The temperature was selected to correspond with those used in the ASTM C672 test method. As a programmable environmental chamber was not used for this test, the thawing temperature corresponds with the room temperature. The cycles were performed with no control of the heating or cooling rate. In other words, containers were removed from the freezer for thawing and placed back in for freezing. As the rate of cooling is not controlled, a temperature logger was placed in the freezer to monitor the temperature profile which is shown in Figure 6. Samples were left in the freezer at −18 °C over the weekends resulting in a total of 5 completed cycles per week. The samples were tested in a semi-saturated condition where the bottom of the samples were submerged in 10 mm or 50 mm of solution (Figure 7). Twelve 150 mm cube samples and twelve cylinder samples were tested with 10 mm of solution. Additionally, twelve cylinders were tested with 50 mm of solution (Method 5). All containers were covered with plastic wrap during freezing and thawing to minimize evaporation. After every 5 cycles, the samples were removed from their containers at approximately 8 h into the thawing cycle. This allowed the solution to thaw sufficiently so that no ice remained. The samples were washed and brushed lightly to dislodge any scaled off material. After washing, the samples were dried at 23 °C at 50% RH for one hour, then weighed. The mass loss was calculated and presented as a percentage of the original mass of the samples. The salt solutions were then replaced and the samples returned to their containers before the start of the next freezing cycle. At the end of 15, 30, and 50 cycles, four cylinder samples were to be tested for compressive strength. Testing was concluded at 50 cycles as suggested in ASTM C672 for conventional concrete.

**Figure 8:**
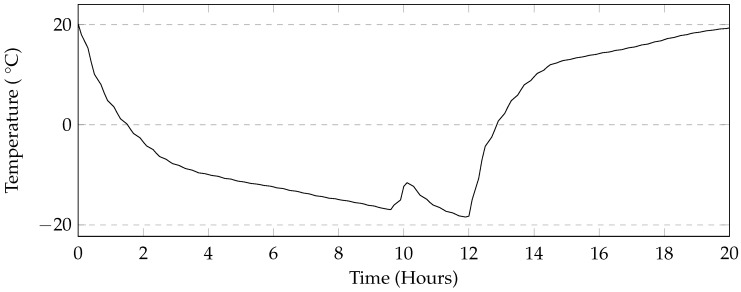
Typical air temperature profile of the environmental chamber; peak is due to defrost system of the machine.

In addition to the above testing condition, a partially saturated test was carried out using a programmable environmental chamber (Method 6). The air temperature inside the chamber varied between 21 °C to −18 °C. The chamber required approximately 12 h to lower the temperature down to −18 °C and approximately 2 h to raise the temperature from −18 °C up to 21 °C (Figure 8). The temperature change would begin every 12 h, therefore one cycle would require 24 h to complete. In this test, cylinder samples were placed in 10 mm of solution. Mass measurements were made every 7 cycles as the automation allowed cycles to occur over the weekends as well. At the end of 14, 35, and 56 cycles, four cylinder samples were tested for compressive strength. The deicers tested under these conditions included CaCl_2_, NaCl, MgCl_2_, and CMA.

#### 2.3.5. 24-h Cycle under Drained Condition (Method 7)

Another set of twelve cylinder samples were tested following the same procedure in Section 2.3.4, but were tested without brine solution while in the freezing part of the cycle. Initially, samples were soaked in CaCl_2_ solution at room temperature for 12 h. After that, samples were allowed to drain for 5 min prior to being placed into the freezer for 12 h (for the freezing cycle). At the end of the freezing period, samples were removed from their containers and placed in solution to start the thawing cycle.

#### 2.3.6. Capacity of the Test Method to Differentiate between Different Deicers

The effects of different deicers on pervious concrete was evaluated using the 24-h cycle partially saturated test. The test procedure is repeated as described in Section 2.3.4, substituting the CaCl_2_ solution for the deicer of interest. In this study, the additional deicers include NaCl, MgCl_2_, urea, potassium acetate, sodium acetate and CMA. All deicing solutions were kept at a concentration of 4% by mass and were replaced every 5 cycles. The 24-h partially saturated cycles was chosen to test different deicers as this was the condition that produced measurable damage when tested with CaCl_2_ solution.

## 3. Results

### 3.1. Drained 12-h Cycle

**Figure 9:**
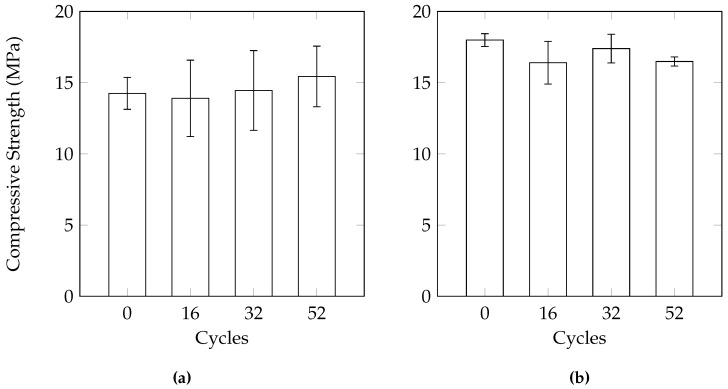
Change in compressive strength under the 12-h drained condition. (**a**) 100 mm cubes; (**b**) Cylinders.

Both the cylinder and the 100 mm cube samples showed minimal amounts of mass loss. An average of 0.45% for the cubes and 0.86% for the cylinders after 52 cycles. The mass loss is mainly a result of the removal of small pieces of cement. The 100 mm cube samples did not show any loss of compressive strength (Figure 9). The cylinder samples showed a statistically significant reduction in compressive strength of only 0.5 MPa after 52 cycles (determined using a one-tailed *t*-test at a 95% confidence level). While this method seems more representative of field conditions, the minimal levels of damage make it difficult to differentiate between the resistance of different pervious concrete mixes to salt scaling. This is likely due to the reduced degree of saturation during the cycles. As the samples were only removed and submerged once every 4 cycles (48 h), it is possible the samples dried out during the third and fourth cycles. This would result in little damage as the level as damage tends to increase with the level of saturation. If the samples were submerged in solution while freezing, this would have produced more damage.

### 3.2. Partially Saturated 12-h Cycle

**Figure 10:**
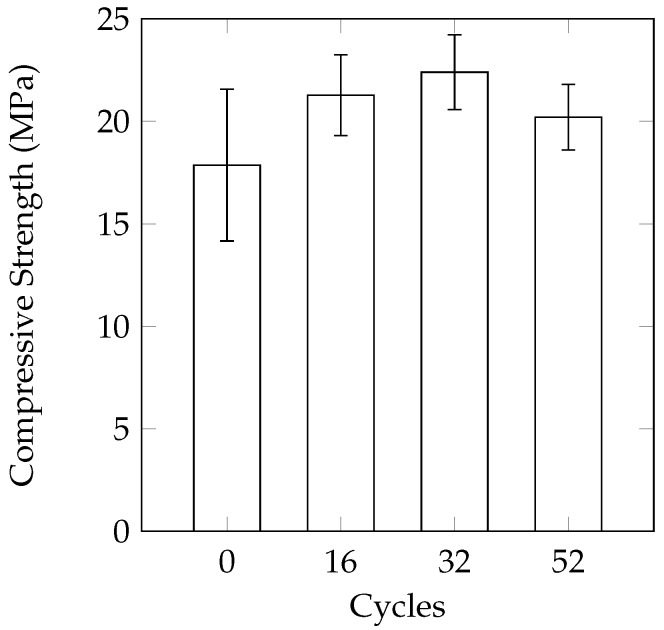
Change in compressive strength under the 12-h partially saturated condition.

The cylinders tested using this testing regime had an average mass loss of 0.45% after 52 cycles. The samples also did not show any reduction in compressive strength after 52 cycles (Figure 10). As a large volume of solution was required to maintain the level at 50 mm, the duration of the thawing phase was not sufficient to completely thaw the entire container. As a result, the authors believe that complete cycles did not occur, resulting in less damage. Since complete freezing did not take place due to the large volume of solution, it is likely that the high relative humidity, due to the presence of the deicing solution, served as additional curing to the samples. This could be the reason behind the slight increase in strength, especially after 32 cycles. However, it should also be emphasized that variability within the samples should be considered when determining change in compressive strength. Indeed, using a *t*-test at a 95% confidence interval, the increase in strength was found to be not statistically significant at 32 cycles.

### 3.3. Partially Saturated 16-h Cycle

**Figure 11:**
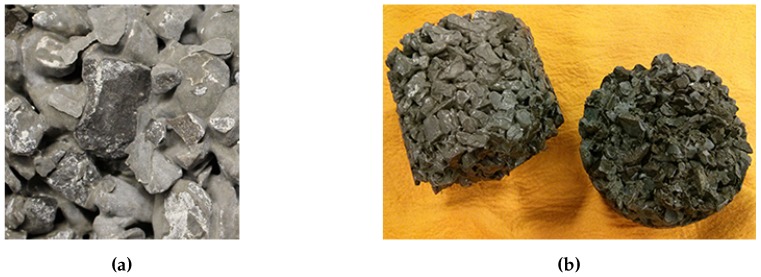
Damage to samples subjected to the 16-h partially saturated cycle. (**a**) Cement stripped off the surface of an aggregate; (**b**) A cylinder sample split in half after 54 cycles.

**Figure 12:**
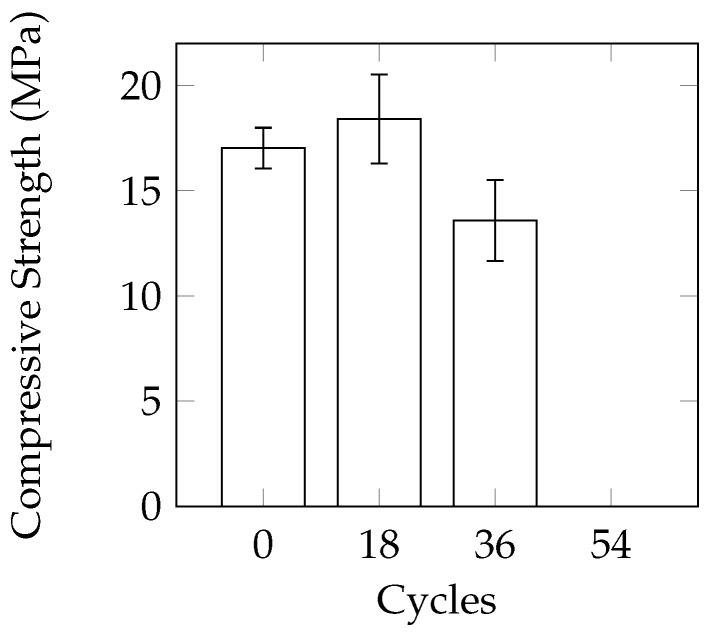
Change in compressive strength under the 16 h partially saturated cycle.

This method was selected to ensure complete freezing and thawing of the solution. This was achieved by: (a) using cylinder moulds to minimize the amount of solution needed to submerge the samples; and (b) lengthening the thawing period. The mass loss was again in the form of small pieces of cement scaling off of the sample. In Figure 11a, an exposed aggregate can be seen as the cement has scaled off from the surface. While it appeared that the mass loss was minimal after 48 cycles (average of 0.67%), the three remaining samples split in half after 54 cycles (Figure 11b). It appears as though the damage was concentrated near the mid-height of the sample, which was also the height of the mould and CaCl_2_ solution. There appears to be a statistically significant (at a 95% confidence interval using a one-tail *t*-test) reduction in compressive strength after 36 cycles (Figure 12).

### 3.4. Partially Saturated and Drained 24-h Cycle

**Figure 13:**
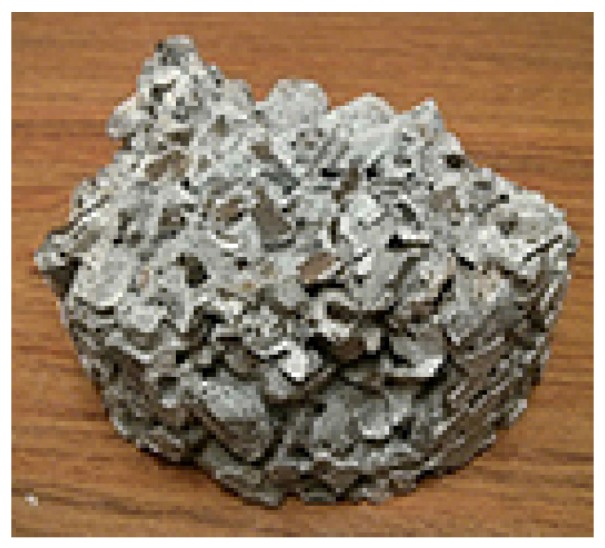
A large intact piece of pervious concrete broken off from the bulk of the sample while being tested with the 24 h cycle saturated with 50 mm of solution.

**Figure 14:**
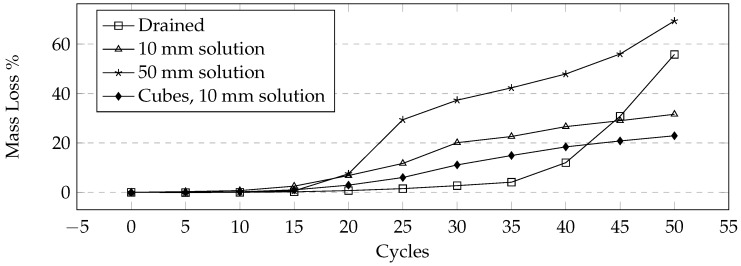
Comparison of average mass loss for different sample shapes and levels of saturation under the 24-h manual cycle using CaCl_2_ solution.

**Figure 15:**
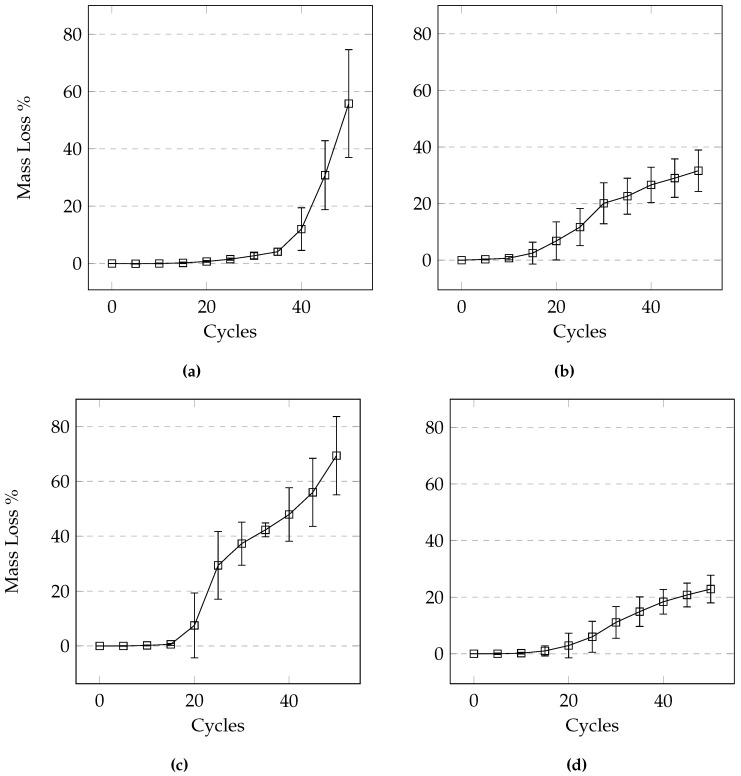
Mass loss comparison of samples under the 24-h manual method using CaCl_2_. (**a**) Drained; (**b**) 10 mm solution; (**c**) 50 mm solution; (**d**) Cubes, 10 mm solution.

This 24-h cycle was used in order to make sure that complete freezing and thawing took place in the samples. The mass loss after a low number of cycles was similar to the previous tests where small cement flakes would scale off. However, as the test progressed, it was apparent that the quantity of mass loss was much higher. As the pervious concrete lost the thin layer of cement, pieces of aggregate would break off from each sample. This resulted in much higher mass loss when compared to earlier tests as the samples suffered a more significant loss of cement paste and was unable to resist the forces generated by the formation of ice. The compressive strength for many samples could not be tested due to significant mass loss. A comparison of different levels of saturation shows a large variance. While gradual at first, the samples tested under 50 mm of solution showed jumps in mass loss due to large pieces breaking off (Figure 13). Comparing the mass loss of 150 mm cubes and the cylinders shows that the general trend between the two types of samples are similar (Figure 14 and Figure 15).

### 3.5. Partially Saturated Cycle Using a Programmable Environmental chamber

Using this testing regime, the average mass loss of the samples in the CaCl_2_ solution after 49 cycles was 5.26%. This is much lower than the 31.64% mass loss at 50 cycles obtained using the same cycles but by moving samples manually to and from the freezer. Testing was continued to 98 cycles due to unexpected low levels of damage. However, even at 98 cycles the average mass loss was only 25.2%. The possible reasons for the reduced damage is discussed in the discussion subsection of this paper.

### 3.6. Effects of Different Deicers

**Figure 16:**
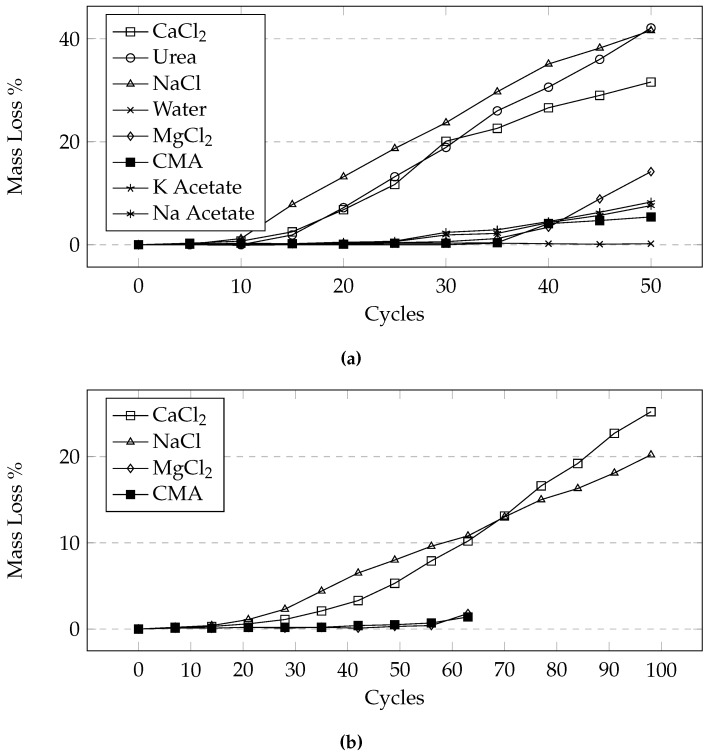
Average mass loss comparison of samples exposed to different deicers under the 24-h cycle using (**a**) the manual method and (**b**) the automatic method.

**Figure 17:**
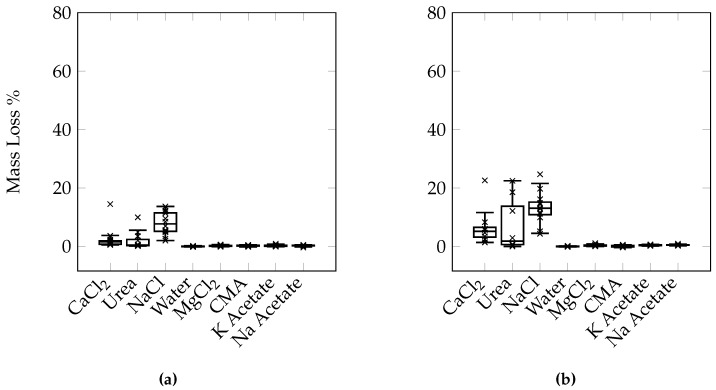

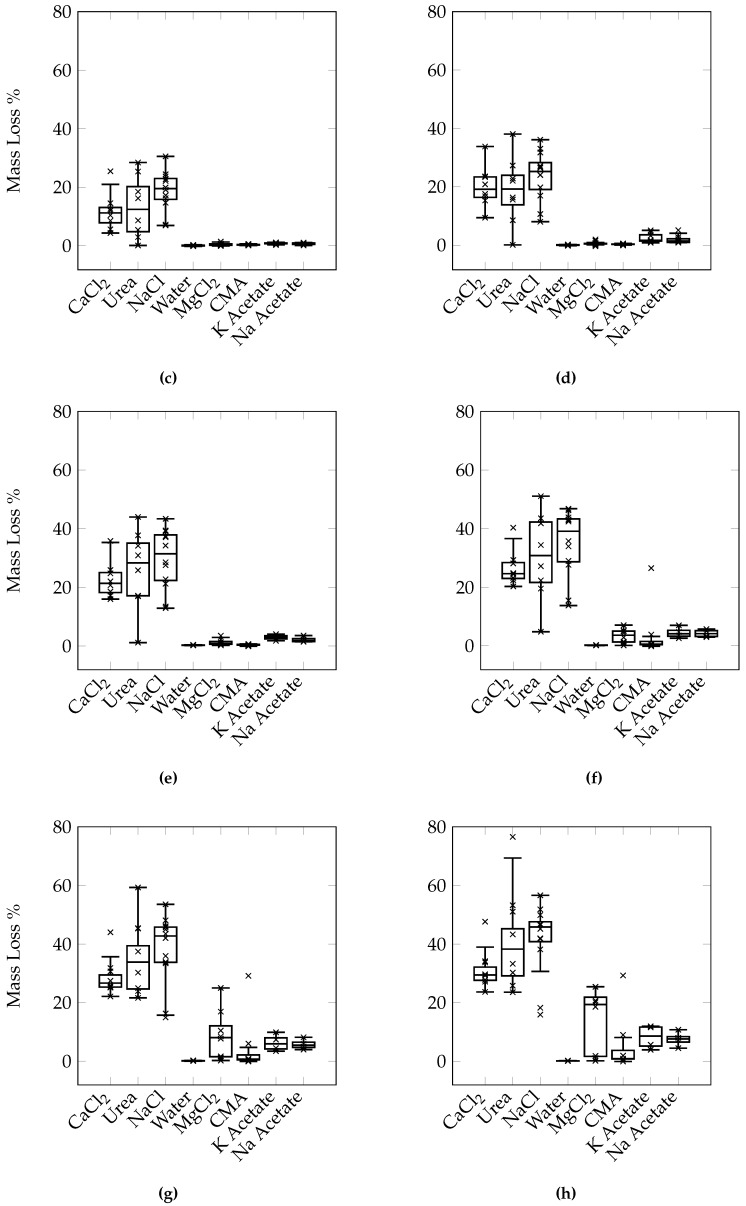
Box plots comparing the mass loss of samples exposed to different deicers under the 24-h manual test. (**a**) 15 cycles; (**b**) 20 cycles; (**c**) 25 cycles; (**d**) 30 cycles; (**e**) 35 cycles; (**f**) 40 cycles; (**g**) 45 cycles; (**h**) 50 cycles.

From Figure 16 we can see that at the same concentration, NaCl and CaCl_2_ cause the most damage followed by MgCl_2_ and CMA. While it appears that there is a difference in the level of damage between NaCl and CaCl_2_, it is not statistically significant (difference between the deicers was determined using the Tukey-Kramer test at a 95% confidence level). Figure 17 illustrates the variability within each set of samples. It is apparent that the variability increases with an increasing number of cycles. It can be seen that the damage develops much earlier on in samples exposed to CaCl_2_, urea, and NaCl when compared to samples exposed to the other deicers.

**Figure 18:**
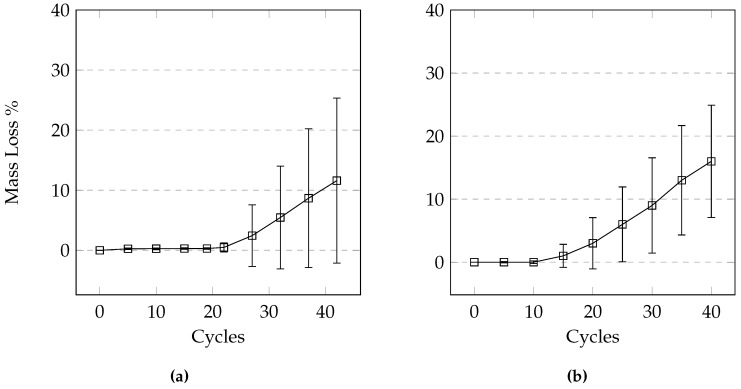
Mass loss comparison of test conducted using 150 mm cubes at ITC Lafarge Canada. Samples exposed to solutions of (**a**) CMA; and (**b**) CaCl_2_.

Since CMA produced low levels of damage, it was decided to confirm the results by repeating the test. The 24-h cycle test was performed at Lafarge Innovation and Training Centre (ITC), Lafarge Canada using 150 mm pervious concrete cubes submerged in 10 mm of CaCl_2_ and CMA deicing solutions. It should be noted that the variability of the test results carried out at Lafarge was slightly higher than the variability of the test carried out at Ryerson. This could be the result of the inherent variability of pervious concrete itself. Figure 18 shows the results of testing CaCl_2_ compared to CMA. It appears that the damage caused by CaCl_2_ is greater than the damage caused by CMA, agreeing with the results obtained at Ryerson’s lab. Although the variability of the mass loss makes it difficult to draw a definitive conclusion, it is apparent that the damage develops much earlier in the samples exposed to CaCl_2_; significant damage occurred by 15 cycles for CaCl_2_ and by 27 cycles for CMA.

## 4. Discussion

### 4.1. Sample Geometry

A comparison of the three different sample geometries (100 mm cube, 150 mm cube, 100 mm diameter by 150 mm height cylinders) does not reveal a large difference between variability of samples in terms of mass loss. The consistency of the compressive strength results of the cylinders was better than the 100 mm cubes. In the study by Cutler et al. [15], the use of 100 mm cubes also resulted in fairly inconsistent compressive strengths. The use of 150 mm cube samples in the current work did not provide a significant improvement over the cylinders in terms of consistency. As freezer space can be a limiting factor in conducting laboratory experiments, the smaller size of the cylinders will allow more samples to be tested at the same time. The reduced size also allows for easier casting and handling of the samples.

### 4.2. Depth of Submersion

For samples that were partially submerged, the damage appeared to be concentrated near the top of the solution; the scaled off cement flakes were lost mainly at this height in the sample. A large part of this mass loss would not necessarily be due to salt scaling damage as much of the cement had not been scaled off. This sudden failure, where large pieces of concrete break off, also occurred in samples in the drained condition. While the damage in the drained condition appeared to be slow and minimal at first, the damage was concentrated at a certain height of the sample, leading to the sudden significant increase in mass loss. It should be noted that the samples were drained of excess liquids, but were not completely dried when frozen. There is a possibility that the failure occurs at the level where the paste was in a saturated surface dry condition. A submersion level of 10 mm seems appropriate for pervious concrete samples made with 14 mm limestone coarse aggregate since the tests show that the damage occurs mainly at the height of the solution. It is possible that pervious concrete made with larger aggregates will not suffer as much damage in this test as the level of solution might be in the mid-height of the stone and would not cause much scaling. Using 10 mm of solution is recommended as the damage occurs gradually rather than in significant jumps.

In conventional concrete the pond of solution on the concrete surface is necessary for salt scaling to occur [2]. Damage still occurred in the drained test in this study, as well as the study by Anderson and Dewoolkar [1] where mass loss exceeded 15% in some cases. It is possible that the solution adhering to the cement paste in pervious concrete is sufficient to act as a pond of solution given the thin layer of paste surrounding the aggregate. It is likely that the same mechanism responsible for salt scaling in conventional concrete is also responsible for salt scaling in pervious concrete. It should be noted that the mechanism of damage is not the main objective of this study.

### 4.3. Automatic and Manual Cycles

**Figure 19:**
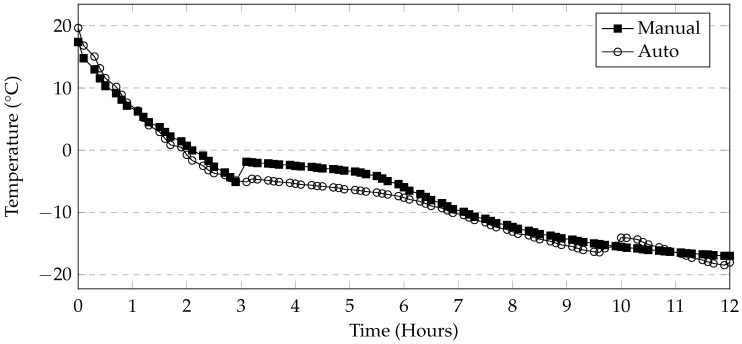
Comparison of deicing solution temperature. Temperature spike likely due to freezer defrost system.

The programmable environmental chamber used in this study provided a rate of cooling of the deicing solution that matched that of the industrial freezer used in this study (Figure 19). This makes it likely that the rapid mass loss seen in the manual methods is attributed to moving the samples to and from the freezer. It is likely that the movement of the samples will cause the specimens to fall over. The subsequent repositioning of the specimen in the solution will allow more damage to occur. Additionally, the samples in the manual method are washed once every 5 cycles, whereas in the automatic cycle, they are washed once every 7 cycles. This is because the use of the automatic or programmable environmental chamber enables testing of the samples over the weekend. Washing would cause much of the material still adhering to the sample to dislodge resulting in exposing new parts of the specimen to the salt solution. The sets of samples tested using MgCl_2_ and CMA in this method also show low levels of damage when compared to the manual method. The general trends in mass loss caused by the different deicers appears to be the same in both cases. A programmable environmental chamber would likely result in better consistency and repeatability.

### 4.4. Cycle Length

Extending the freezing period appears to cause more damage. In the case where samples were partially saturated, the extension of the freezing period allowed the solution to reach a lower temperature. The 12-h freezing period of the 24-h cycles was sufficient to bring the temperature of the solution down to the minimum temperature of −18 °C. The long 12-h thawing period was required to allow both thawing and washing of the samples. As the entire washing and weighing procedure can take up to four hours, the solution requires enough time to ensure complete thawing by the time the washing procedure begins. Based on this, the 24-h cycle is recommended for testing previous concrete.

### 4.5. Different Deicers

While a conclusive order of the relative levels of damage of each deicer cannot be concluded from the results of this study, a general trend can be formed. CaCl_2_, NaCl, and urea appear to be the most damaging, MgCl_2_, K acetate, Na acetate, and CMA are less damaging, but all deicers cause more damage than freezing/thawing in water alone. The difference between the various deciers can be due to a number of reasons. It should first be noted that although all the salts are at the same concentration by mass percent, the molality of each is different. As molality is calculated by dividing the number of moles of the solute by the mass of the solvent, an equal mass of solute would result in a higher molality for a solute with a lower molecular weight since there would be more moles of that solute at an equal mass. Additionally, the resulting freezing point depression is also different for the different deicers. The molality of both the NaCl and MgCl_2_ solutions is higher than CaCl_2_, while the freezing point of both is lower. However, the results indicate that while NaCl potentially causes more damage than CaCl_2_, MgCl_2_ does not. If either of these factors alone correlate with the damage, NaCl and MgCl_2_ should both cause either more damage or less damage than CaCl_2_.

From the reported literature, CaCl_2_, MgCl_2_, and CMA often cause more severe damage than NaCl through chemical deterioration after long term exposure [6]. For a number of deicers in this test, the damage was apparent as early as ten cycles.

While the results of the study by Cutler et al. [15], showed that CaCl_2_ caused more damage than NaCl, the concentration of each deicer was at 9%. Therefore it cannot be said that the results of the present study contradicts the results presented by Cutler et al. [15]. In conventional concrete, at the pessimum concentration, it has been shown that NaCl causes more damage than CaCl_2_ [2]. While at higher concentrations, Wang et al. [12] found that CaCl_2_ causes more salt scaling damage than NaCl, similar to Cutler et al. [15].

It should be reemphasized that the use of different salts in this research was mainly to evaluate the possibility of the optimized test method with different salts. The long term effects of different salts on pervious concrete under different temperatures was not investigated here.

### 4.6. Failure Criteria

**Figure 20:**
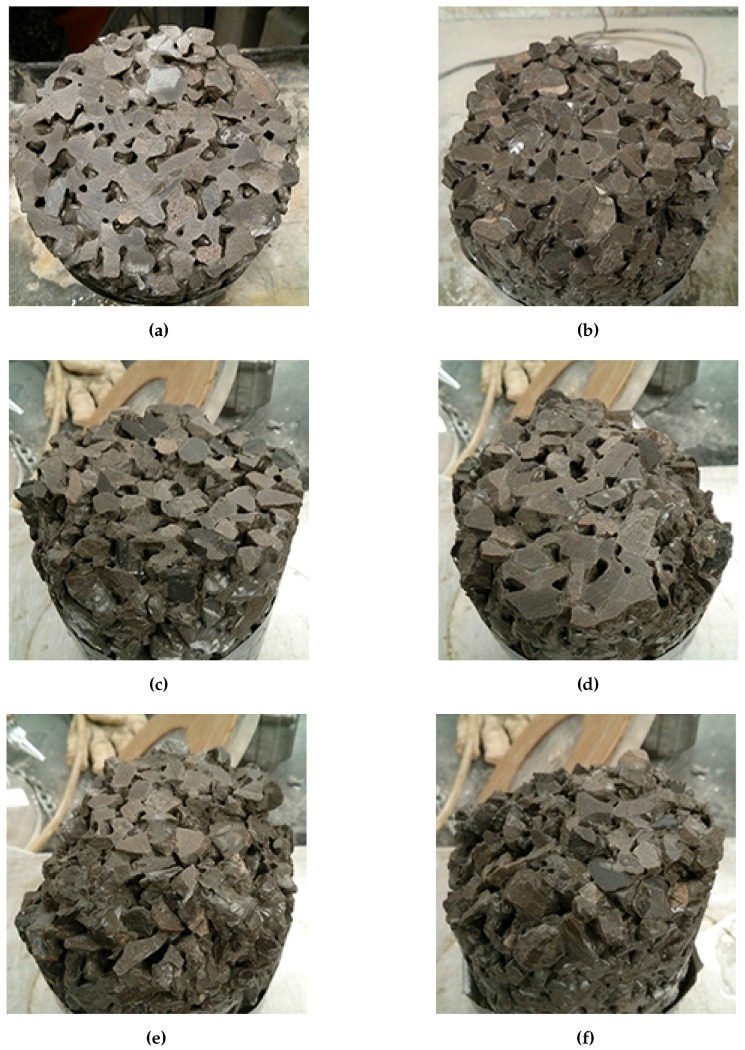
Various levels of mass loss of cylindrical samples. (**a**) 0%; (**b**) 1.0%; (**c**) 2.5%; (**d**) 4.7%; (**e**) 6.0%; (**f**) 8.3%.

Based on a visual inspection of the remaining flat surface area of each sample compared to its mass loss, a cutoff point can be determined. For a cylinder sample with a diameter of 100 mm and a height of 150 mm, a mass loss in the range of 3%–5% is suggested based the remaining area of the initial cut surface (Figure 20). Most samples are still able to stand upright in this range, however, it is apparent that severe damage has occurred. The ability of the samples to stand up can be taken as an indication of adequate contact or load transfer between the pervious concrete and the underlying granular material, assuming that the damage occurs at the bottom of the concrete layer. If scaling occurs at the top of the pervious concrete, 3%–5% mass loss represents a pervious concrete layer that is still reasonably intact.

Using this criteria, CaCl_2_, urea, and NaCl exceeded 3%–5% mass loss within 30 cycles. The exact number of cycles is difficult to determine as the mass was taken only once every 5 cycles. Additionally, this occurs at different times for different specimens of the same set. It is suggested that the average or the median mass loss within each set of samples be used to determine the number of cycles to failure. Due to the variability present, the use of a median mass loss may be more appropriate. Using this, samples exposed to solutions of CaCl_2_, urea, and NaCl failed after 20, 25, and 15 cycles respectively. Samples exposed to solutions of MgCl_2_, K acetate, and Na acetate would all be considered to have failed at either 40 or 45 cycles, depending on whether the upper or lower limit of the criterion is selected. Samples exposed only to water did not show any signs of damage, confirming that the damage is mainly due to the use of salts. Although the use of the median value indicates that CMA will not cause sample failure even after 50 cycles, a number of individual samples did suffer particularly high levels of mass loss. It is possible that continued long term use of CMA will eventually lead to severe deterioration. On the other hand, the ability of the pervious concrete to drain water coupled with the exposure of pavements to rain without salts in the summer may help to flush the system and reduce the accumulation of deicers within the concrete and its associated chemical attack. Field trials should be conducted to better correlate the test results to in field performance.

## 5. Conclusions

Based on the materials tested in this study, the main conclusions are as follows:

- A cycle length of 24 h (12 h freeze and 12 h thaw) was found to be optimum in terms of ensuring complete freezing and thawing. A saturation level of 10 mm is suggested to cause gradual but significant mass loss.
- Cylinder samples were found to be easy to cast and handle and can also provide consistent compressive strength and mass loss results; however, 150 mm cubes provided similar results.
- At an equal concentration by mass (4%), the most severe salt scaling damage was caused by CaCl_2_, NaCl, and urea. MgCl_2_, K acetate, Na acetate, and CMA did not cause as much damage, and all deicers caused more damage than freezing and thawing in water alone. However, the long term effects of these salts on concrete were not investigated in this study and should be considered when comparing different deicers.
- The use of an automatic or programmable environmental chamber resulted in less damage compared to manually moving samples in and out of a freezer. The general trend of the relative levels of damage of the different deicers remained the same in either the manual or automatic method.
- For cylinder samples with a diameter of 100 mm and a height of 150 mm, a failure criteria of 3%–5% mass loss is suggested.

## Figures and Tables

**Table 1:** Cementing material chemical composition.

Cementing	SiO_2_	Al_2_O_3_	Fe_2_O_3_	TiO_2_	CaO	MgO	SO_3_	S^2−^	LOI
Material	(%)	(%)	(%)	(%)	(%)	(%)	(%)	(%)	(%)
GU	19.6	4.9	3.1	-	61.4	3	3.6	-	2.3
GGBFS	38.4	10.61	0.79	0.71	34.2	6.94	0.2	1.1	3.09

**Table 2:** General mix properties.

w/cm Ratio	Cement Type	GGBFS Replacement (%)	Coarse Aggregate	Coarse Aggregate Fraction	Fine Aggregate Fraction
0.3	GU	15	CSA certified 14 mm	1	0
			crushed limestone		

**Table 3:** Summary of various testing parameters used for the different tests.

Method	Automated Cycles	Cycle Length (Hours)	Temperature (°C)		Submersion Level (mm)	Deicers Used
			High	Low		
1	Yes	12	10	−18	0	CaCl_2_
2	Yes	12	10	−18	50	CaCl_2_
3	Yes	16	15	−18	75	CaCl_2_
4	No	24	21	−18	10	CaCl_2_ for cubes,
						all deicers for cylinders
5	No	24	21	−18	50	CaCl_2_
6	Yes	24	21	−18	10	CaCl_2_
7	No	24	21	−18	0	CaCl_2_
ASTM C672	No	24	23 ± 2	−18 ± 3	-	CaCl_2_
Conventional						
Concrete						

## Abbreviations

The following abbreviations are used in this manuscript:

CMA	Calcium magnesium acetate
KA	Potassium acetate
NaA	Sodium acetate
